# Health-seeking behaviors of pregnant women with hypertensive disorders of pregnancy in low- and middle-income countries: A scoping review protocol

**DOI:** 10.1371/journal.pone.0357049

**Published:** 2026-08-27

**Authors:** Mariam Adam Al-Hassan, Jodie Bigalky, Solina Richter

**Affiliations:** College of Nursing, University of Saskatchewan, Saskatoon, Canada; Freelance Consultant, Myanmar, MYANMAR

## Abstract

Hypertensive disorders of pregnancy (HDP) are among the leading causes of maternal and perinatal illness and death worldwide. Despite advances in its management, the burden of HDP remains disproportionately high in Low- and Middle-Income Countries (LMICs), where issues related to healthcare access, cultural beliefs, and systemic inefficiencies hinder timely diagnosis and intervention. Health-seeking behavior is crucial for preventing severe complications linked to HDP. An initial review of the literature was conducted and revealed no scoping or systematic reviews on the health-seeking behaviors of pregnant women with HDP in LMICs. We outline a scoping review protocol, using the JBI methodology, to map and synthesize existing evidence on the barriers and facilitators that influence the health-seeking behaviours of pregnant women with HDP in LMICs. The protocol is registered with the Open Science Framework (https://doi.org/10.17605/OSF.IO/FTSR5). The data search and screening were completed on May 29 and June 28, 2025, respectively. Data extraction is ongoing. Study results and analysis are expected by December 2025, with journal submission and publication planned for January 2026. Findings will be valuable in providing a comprehensive overview of the current landscape of health-seeking behaviours, identifying gaps, and guiding appropriate care outcomes across settings in LMICs.

## Introduction

Globally, maternal and perinatal mortality remains a significant public health concern. Hypertensive disorders of pregnancy (HDP) rank among the leading causes of maternal and perinatal morbidity and mortality, with a worldwide prevalence of 116.4 per 100,000 women of childbearing age [1–3], accounting for approximately 14% of all maternal deaths [4]. Over the years, recommendations regarding using and selecting antihypertensive medications to prevent and treat hypertension during pregnancy have aimed to improve the quality of care and outcomes for pregnant women [3,4]. Consequently, mortality and incidence rates have decreased in most countries, particularly in high-income countries (HIC) [1].

The Society of Obstetrics and Gynecology of Canada (SOGC) defines hypertension in pregnancy as a systolic blood pressure of at least 140 mmHg and a diastolic blood pressure of at least 90 mmHg when measured in a clinic or office setting [5]. Hypertensive disorders of pregnancy (HDP) encompass a variety of conditions characterized by elevated blood pressure during pregnancy [5,6]. The SOGC identifies chronic hypertension, gestational hypertension, chronic hypertension with superimposed preeclampsia, and preeclampsia and eclampsia as HDP [5]. The SOGC guidelines emphasize identifying high-risk women as early as possible, implementing preventative measures such as lifestyle modifications, and maintaining diligent observation throughout the pregnancy. Treatment encompasses medication and monitoring alongside blood pressure control; in some instances, early delivery is necessary to prevent complications [5].

Despite these recommendations, the burden of HDP remains disproportionately high in low- and middle-income countries (LMICs) [1,2,7]. Africa, Southeast Asia, and the Middle East have prevalence rates of 334.88, 136.81, and 121.4 per 100,000 women of childbearing age, respectively. In contrast, the Western Pacific region recorded the lowest prevalence at 16.4 per 100,000 women of childbearing age [2].

A comprehensive study on global trends of HDP revealed that areas with a high-middle sociodemographic index (SDI) have reduced Disability Adjusted Life Years (DALYs) associated with HDP [8]. SDI is a composite measure of a region’s socioeconomic development, while DALYs quantify the burden of disease by accounting for both years of life lost due to premature death and years lived with disability. Conversely, low- and middle-income SDI regions have experienced an increase in DALYs, highlighting the disparities in the global burden of HDP [8]. These findings suggest that a rise in SDI correlates with a decrease in the disease’s impact. This reflects the challenges faced by many low SDI countries, which lack sufficient resources to detect, address, and treat HDP effectively. The results underscore the increasing prevalence and emphasize the need for appropriate measures and better resource distribution to tackle the growing challenge of HDP in low SDI countries. Low SDI countries often overlap with LMICs, as both classifications feature regions facing significant economic, social, and health challenges that hinder progress toward achieving critical developmental goals. With less than five years remaining until 2030, efforts to achieve the Sustainable Development Goal (SDG) target of fewer than 70 per 100,000 live births have not yet been realized in LMICs. This highlights the need to intensify actions to address persistent disparities contributing to perinatal morbidities and mortalities.

Early detection, timely health-seeking, and appropriate management of HDP are crucial in reducing adverse perinatal outcomes [3,9–11]. Nonetheless, various barriers to accessing healthcare exist in LMICs, hindering identification and intervention efforts. These barriers include sociocultural and religious factors, difficulties accessing healthcare, financial constraints and a lack of education [12–15]. Additionally, systemic inefficiencies within healthcare systems and issues related to the training and capacity building of healthcare providers further exacerbate the challenges of managing maternal conditions in a timely manner [14–18]. Addressing HDP requires a multisectoral approach across various sectors to achieve SDG 3.1, which aims to reduce maternal mortality [19,20].

Despite the growing concern and focus on maternal health, along with the increasing accessibility of antenatal services in LMICs, timely healthcare utilization among pregnant women with hypertensive disorders remains an issue [3,16]. Health-seeking behavior refers to actions taken by individuals when they suspect a disease or health issue that requires treatment [21]. We conceptualise health-seeking behaviours as actions that facilitate timely and effective care, such as early antenatal care, adequate attendance, prompt care-seeking for symptoms as appropriate or positive, and inappropriate (negative) behaviours that may delay care or hinder appropriate care (e.g., delayed presentation, reliance on non-formal care when formal care is required). These behaviors are complex and shaped by knowledge, beliefs, cultural practices, socioeconomic status, and access to health services and healthcare systems [22]. However, health behaviors can change if the barriers are clearly identified and adequately addressed with the appropriate support and interventions. Health-seeking behaviors will be anchored in the socio-ecological model (SEM) in this review. SEM acknowledges the complex, multilevel influences shaping behavior [23]. Guided by this framework, the review will systematically organize health-seeking behaviors across different social levels to map the existing evidence and generate insights that may inform targeted interventions.

For pregnant women experiencing HDP, timely recognition of symptoms and prompt health-seeking can prevent severe complications. However, care is often sought late, which increases the risk of adverse outcomes for both the mother and the fetus [24]. Understanding the patterns, barriers, and facilitators of health-seeking behaviours among this population is essential for developing targeted interventions to improve outcomes. Existing reviews have focused on the health-seeking behaviors of pregnant adolescents, and management and experiences of pregnant women facing HDP [15,25,26]. However, evidence regarding the health-seeking behaviors of pregnant women with HDP remains to be comprehensively mapped. This protocol contributes to the literature by outlining a systematic approach to mapping existing evidence on both barriers and facilitators influencing health-seeking behaviors across the care continuum for HDP. Additionally, capturing evidence for both appropriate and inappropriate health-seeking behaviors from pre-diagnosis through outcomes provides a comprehensive understanding of care-seeking pathways.

A scoping review is necessary to explore what is known about health-seeking behaviors of women with HDP and to identify barriers and facilitators to care. The findings may contribute to informing culturally appropriate interventions, offering recommendations to improve the quality of care for women with HDP, and highlighting implications for reducing adverse perinatal outcomes. Furthermore, the review will synthesize existing knowledge, identify research gaps, and inform future investigations. Consequently, the review will comprehensively examine the literature on the health-seeking behaviors of HDP across LMICs, with a focus on barriers, facilitators, existing gaps, and reported outcomes. This scoping review is the initial stage of a multiphase project and informs a subsequent study in Ghana that will use a critical ethnographic approach aimed at exploring context-specific determinants of health-seeking behaviors and generating locally relevant evidence to guide interventions.

## Materials and methods

This review will employ the Joanna Briggs Institute (JBI) methodology, utilizing the Preferred Reporting Items for Systematic Reviews and Meta-Analysis extension for Scoping Reviews (PRISMA-ScR) [27,28]. The protocol is registered with the Open Science Framework (https://doi.org/10.17605/OSF.IO/FTSR5).

### Research question(s)

What are the health-seeking behaviors of pregnant women with HDP in LMICs?

### Inclusion and exclusion criteria

The following criteria will be included in the review, while all others will be excluded.

#### Participants.

This review evaluates studies on the health-seeking behaviors of pregnant women with HDPs in LMICs. These include studies examining health-seeking behaviors in relation to HDP, regardless of whether individuals were diagnosed, undiagnosed, or experienced mortality related to HDP. Specifically, studies will be included if they involve individuals with a confirmed diagnosis of HDP, explore care-seeking behaviors through which HDP may be detected, or examine health-seeking behaviors that may influence early identification, management, or outcomes of the condition. Pregnant women are individuals carrying a developing fetus within their uterus from conception until birth. This period lasts approximately 40 weeks and is divided into three trimesters, during which various changes occur to support fetal growth, development and delivery.

#### Concepts.

HDP is a group of conditions marked by high blood pressure (at least 140/90 mmHg) occurring during pregnancy. HDP includes chronic hypertension, gestational hypertension, chronic hypertension with superimposed preeclampsia, preeclampsia and eclampsia.

#### Health-seeking behavior.

Health-seeking behavior refers to the actions individuals take to maintain, restore, or improve their health, which include recognizing symptoms, seeking medical attention, adhering to treatments, and utilizing healthcare services. These behaviors are influenced by knowledge, cultural beliefs, socioeconomic status, and access to healthcare, among others. HDP are a group of conditions that cause high blood pressure during pregnancy.

#### Context.

This study examines women’s health-seeking behaviors related to HDP across 109 LMICs worldwide, focusing on urban and rural settings. This figure is derived from World Bank classifications, categorizing economies into four income groups: low, lower-middle, upper-middle, and high. In this context, LMICs are categorized into three groups: low-income, lower-middle-income, and upper-middle-income countries. The classification serves analytical purposes using gross national income (GNI) per capita data in U.S. dollars [29,30].

### Types of sources

This scoping review will consider non-peer-reviewed and published evidence. Quantitative, qualitative, and mixed-method studies will be considered irrespective of their methodological approach. Commentaries and conference abstracts will also be considered for inclusion. Electronic databases, reference lists, and hand-searching will be utilized to gather literature on published studies. The focus will be on nursing, allied health, biomedical, social sciences, regional databases, and sources of grey literature.

### Search strategy

The search strategy, which included both an initial and secondary search, was conducted in partnership with a research librarian. The search targets both published and unpublished studies. A comprehensive search strategy was developed for the concepts and population using keywords from articles and abstracts of relevant articles [31,32], along with controlled vocabulary such as Medical Subject Headings (MeSH).

MEDLINE was chosen because of its comprehensive and authoritative database of biomedical and health sciences literature, which ensures a thorough and systematic identification of existing literature. For each database and information source included, we adapted the search strategy to incorporate all identified keywords and index terms using the Polyglot translator [33]. The following databases were used to search for published literature on May 28, 2025: Medline (Ovid), Embase (Ovid), Cumulative Index of Nursing and Allied Health Literature Plus (CINAHL) (EBSCOhost), Scopus (Elsevier), and APA PsycInfo (Ovid).

Grey literature was obtained from the ProQuest Dissertations and Theses Global database, Global Index Medicus (GIM interface), FIGO, JHPIEGO, the World Health Organization (WHO) website, and the United Nations Population Fund (UNFPA) website on May 28, 2025. An advanced Google search was conducted in incognito mode on May 28, 2025, to eliminate the impact of previous search histories on the results. The following keywords were used: “hypertensive disorders of pregnancy”, “health-seeking behavior”, and “low and middle-income countries”.

No date restrictions were applied to ensure a comprehensive capture of all relevant literature, regardless of publication year. Although multiple languages are spoken across the regions, this review will include only articles published in English. This decision is based solely on the language proficiency of the review team and is intended to ensure accuracy during data extraction and interpretation. We acknowledge that restricting the search to English-language studies may result in the exclusion of relevant evidence and therefore represents a limitation in this review.

The search strategies for Medline and grey literature can be found in the Supporting Information (S1 File) and (S2 File), respectively. After the search, the results from each database were documented and saved. Citations were imported into Mendeley, a bibliographic management tool, and duplicates removed. The remaining citations were then be uploaded to Covidence, a software system used by reviewers for managing, screening and extraction. Two independent reviewers evaluated the titles and abstracts based on the established inclusion and exclusion criteria

Two independent reviewers reviewed only relevant papers in full text, and one will extract data from all included papers. The reasons for exclusion will be noted and documented in the final report. Disagreements at any stage of the screening process will be resolved by involving a third reviewer and/or discussion between the reviewers. These disagreements will serve as an opportunity to strengthen the screening process. The PRISMA-ScR flow diagram will be used to present the final search results [28].

### Data extraction

The first data searches were completed on May 29, 2025, and the results were documented and saved. Two reviewers began screening on June 1 to select articles for full-text review and continued the process of full-text review until June 28, 2025. A single reviewer began data extraction on July 1, 2025, and it is ongoing, with completion expected by the end of August. The data extraction process involves reading the entire article multiple times and examining all sections to extract the necessary information. Covidence and Microsoft Excel will be used for data extraction, analysis and presentation. These include the introduction, methods, discussion, conclusions, and even supplementary information [34].

At the beginning of data extraction, the first two papers (15%) extracted were reviewed to ensure consistency and clarity, and issues were clarified by a second reviewer. Additionally, upon completion of the data extraction, the team members will conduct a review of all extracted data to verify consistency, accuracy, and any identified discrepancies will be resolved through a discussion. The extraction tool can be found in the Supporting Information (S3 File). The study results and analysis are anticipated to be completed by September 2025, and a second data search is planned for October, prior to final closure in December 2025. The aim is to submit the findings to a journal by January 2026. The data extracted, as applicable, include author(s), year of publication, study purpose, study design, characteristics of the target population or sample size, study setting or location, key findings (such as theories, influencing factors, barriers, and facilitators), and study recommendations. Authors of the included articles will be contacted if necessary (i.e., if any data are missing). The final scoping review will specify all changes made after the preliminary protocol.

### Data analysis and presentation

An inductive thematic analysis approach will be used to synthesize and categorize the extracted data. During data extraction, all relevant factors influencing health-seeking behaviors will be identified and coded through repeated review of the included studies. Codes will then be grouped into categories and subsequently organized into broader themes through an iterative process.

Barriers and facilitators will be distinguished based on their reported influence on health-seeking behaviors. Factors described by authors as hindering or negatively affecting care-seeking will be categorized as barriers, while those described as enabling or positively influencing care-seeking will be categorized as facilitators.

For factors not clearly labelled by the study authors, classification will be guided by their direction of effect as reported within the study context. Factors that do not show a clear directional effect or whose influence varies by context will be categorized as neutral or context-dependent and will be analyzed separately in the narrative synthesis.

Furthermore, identified barriers and facilitators will be grouped into domains such as individual-level (e.g., knowledge, perceptions, beliefs, and health literacy), socio-cultural, economic and community factors, such as income, education, location, occupation, financial constraints, cultural beliefs, gender norms, family influence and community practices) and health system factors (e.g., access to care, quality of services, availability of resources, and provider-related issues reflective of the socio-ecological framework. Each identified barrier or facilitator will be assigned to one of these categories as described in the original study; where barriers or facilitators span across multiple domains, it will be acknowledged and reflected in the narrative synthesis. In addition, contextual information, such as the study setting (e.g., country and geographic location), will be extracted and descriptively presented. Variations across geographic context will be explored narratively to highlight patterns, similarities and differences in the reported findings.

Equally, to address contradictory findings, a context-specific approach to data synthesis will be adopted. Contradictory findings will be systematically identified, described and explored within the narrative synthesis. Potential sources of differences will be examined, including differences in the study context such as rural versus urban settings, population characteristics, healthcare system structures and methodological approaches.

All findings will be presented using a combination of tabular and visual formats to improve clarity and accessibility. In addition, a descriptive narrative synthesis will be provided to contextualize and interpret the findings in relation to review objectives, highlighting key barriers and facilitators, stakeholder influences and gaps in the existing literature.

## Supporting information

S1 File Search strategy in Ovid Medline. (DOCX)

S2 File Search strategy for preliminary grey literature. (DOCX)

S3 File Data extraction instrument. (DOCX)

S1 Checklist Preferred reporting items for systematic reviews and meta-analysis for protocols (PRISMA-P, 2020) checklist. (DOCX)
