# Supplementary material for: Health-seeking behaviors of pregnant women with hypertensive disorders of pregnancy in low- and middle-income countries: A scoping review protocol
Source: PLoS One. 2026 Aug 27;21(8):e0357049. doi: 10.1371/journal.pone.0357049 (PMC13521393; doi:10.1371/journal.pone.0357049)
Supplement: Supplementary file 1 [file pone.0357049.s001.docx]

# **S1 File. Search strategy in Ovid Medline**

Database(s): **Ovid MEDLINE(R) ALL** 1946 to May 22, 2025

| **#** | **Searches** | **Results** |
| --- | --- | --- |
| 1 | ((health* or care or help) adj4 seek*).mp. | 54421 |
| 2 | ((acceptance or delay or refus*) adj4 (health* or care)).mp. | 68222 |
| 3 | ((treatment* or program* or support* or services* or health*) adj4 (access* or choices* or barrier* or facilitator* or utilization or utilisation)).mp. | 289365 |
| 4 | hypertension, pregnancy-induced/ or eclampsia/ or hellp syndrome/ or pre-eclampsia/ | 45936 |
| 5 | (Pre-Eclampsia or Preeclampsia or eclampsia or HELLP syndrome).mp. | 61874 |
| 6 | (Hypertensi* adj4 (pregnan* or gestational or maternal)).mp. | 24362 |
| 7 | afghanistan/ or angola/ or bangladesh/ or benin/ or bhutan/ or bolivia/ or burkina faso/ or burundi/ or cabo verde/ or cambodia/ or cameroon/ or central african republic/ or chad/ or comoros/ or congo/ or "cote d'ivoire"/ or "democratic people's republic of korea"/ or "democratic republic congo"/ or djibouti/ or eritrea/ or eswatini/ or ethiopia/ or gambia/ or ghana/ or guinea/ or guinea-bissau/ or haiti/ or honduras/ or exp india/ or jordan/ or kenya/ or kyrgyzstan/ or laos/ or lebanon/ or lesotho/ or liberia/ or madagascar/ or malawi/ or mali/ or mauritania/ or melanesia/ or micronesia/ or morocco/ or mozambique/ or myanmar/ or nepal/ or nicaragua/ or niger/ or nigeria/ or pakistan/ or papua new guinea/ or philippines/ or rwanda/ or samoa/ or "sao tome and principe"/ or senegal/ or sierra leone/ or somalia/ or south sudan/ or sri lanka/ or sudan/ or syria/ or tajikistan/ or tanzania/ or timor-leste/ or togo/ or tunisia/ or uganda/ or uzbekistan/ or vanuatu/ or vietnam/ or yemen/ or zambia/ or zimbabwe/ or ((low* adj3 (income countr* or income nation*)) or LMIC or LMICs or Afghanistan or Afghani or Afghan or Angola* or Bangladesh* or Benin or Beninese or Bhutan or Bolivia* or "Burkina Faso" or Burkinabe or Burundi* or "Cabo Verde" or "Cape Verde" or Cambodia* or Cameroon* or "Central African Republic" or Chad or Chadian or Tchad or Comoros or Comoran or Congo or Congolese or "Cote d'Ivoire" or Ivorian or Djibouti or Egypt or Egyptian* or Eritrea* or Eswatini or Ethiopia* or Gambia or Gambian or Ghana* or Guinea or Guinea-Bissau* or Haiti or Haitian or Hondura* or India or (Indian not American) or Jordan or Jordanian* or Kenya* or Kiribati or "Kyrgyz Republic" or Lao or Laos or Laotian or Lebanon or Lesotho or Mosotho or Basotho or Liberia* or Madagascar or Malagasy or Malawi* or Mali or Malian or Mauritania* or Micronesia* or Morocco or Moroccan or Mozambique or Mozambican or Myanmar* or Burmese or Nepal or Nepalese or Nicaragua* or Niger or Nigerien* or Nigeria* or "North Korea*" or Pakistan* or "Papua New Guinea*" or Philippines or Filipino* or Rwanda* or Banyarwanda or Samoa* or "Sao Tome and Príncipe" or "San Tomean" or Senegal* or "Sierra Leone*" or "Solomon Island*" or Somalia* or "Sri Lanka*" or Sudan or Sudanese or Syria or Syrian or Tajikistan or Tajik or Tadzhik or Tanzania* or "Timor-Leste" or Timorese or Togo or Togolese or Tunisia* or Uganda* or Uzbekistan* or Uzbeki or Vanuatu or Vietnam* or "West Bank" or Gaza or Yemen* or Zambia* or Zimbabwe*).tw,cp. | 1425601 |
| 8 | 1 or 2 or 3 | 375534 |
| 9 | 4 or 5 or 6 | 74238 |
| 10 | 7 and 8 and 9 | 287 |
| 11 | limit 10 to english language | 282 |
