# Supplementary material for: Health-seeking behaviors of pregnant women with hypertensive disorders of pregnancy in low- and middle-income countries: A scoping review protocol
Source: PLoS One. 2026 Aug 27;21(8):e0357049. doi: 10.1371/journal.pone.0357049 (PMC13521393; doi:10.1371/journal.pone.0357049)
Supplement: Supplementary file 2 [file pone.0357049.s002.docx]

# **S2 File. Search strategy for preliminary grey literature.**

| **Date** | **Organization name & website URL** | **Search strategy(s)/ words searched including (if applicable) how items were selected*.*** | **No. items retrieved/ search results** | **No. of items screened uploaded to citation management software** |
| --- | --- | --- | --- | --- |
| 28 May 2025 | ProQuest Dissertations and Theses Global through the University of Saskatchewan library  <https://www.proquest.com>  /pqdtglobal/advanced | (health-seeking behaviors) AND (hypertensive disorders of pregnancy) AND (low- and middle-income countries)  **Language:** English | Items retrieved: 294 | Uploaded to citation management software: **30** |
| 28 May 2025 | Global Index Medicus  <https://www.globalindex>  medicus.net/ | **Search Terms: Hypertensive disorders of pregnancy**  **Major Subject Filters:** Pre-Eclampsia, Hypertension, Hypertension, Pregnancy-Induced, Pregnancy Complications, Maternal Mortality, Eclampsia, Pregnancy Outcome  **Study Type Filters:** Observational studies, Etiology studies, Screening studies  **Collection Filters:** LILACS, IMSEAR, IMEMR, AIM  **Language Filter:** English | Items retrieved: 64 | Uploaded to citation management software: **13** |
| 28 May 2025 | FIGO  <https://www.figo.org/search/node?keys=> | Hypertensive disorders of pregnancy | Items retrieved:  21 | Uploaded to citation management software: 0 |
| 28 May 2025 | UNFPA  <https://www.unfpa.org/search/content?search=> | Hypertensive disorders of pregnancy | Search  results: 12 | Uploaded to citation management software:0 |
| 28 May 2025 | WHO  https://www.who.int/home/search- | Hypertensive disorders of pregnancy | Search results: 600 | Uploaded to citation management software:**1** |
| 28 May 2025 | JHPIEGO  <https://www.jhpiego.org/?s=> | Hypertensive disorders of pregnancy | Search  results: 0 | Uploaded to citation management software: 0 |
| 29 May 2025 | Google Advanced search | filetype:pdf "hypertensive disorders of pregnancy" health-seeking behaviour | Search results: 199 | Uploaded to citation management software: **2** |
| 29 May 2025 | Google Advanced search | filetype:pdf "hypertensive disorders of pregnancy" low and middle-income countries | Search results: 169 | Uploaded to citation management software: 0 |
