# Supplementary material for: Health-seeking behaviors of pregnant women with hypertensive disorders of pregnancy in low- and middle-income countries: A scoping review protocol
Source: PLoS One. 2026 Aug 27;21(8):e0357049. doi: 10.1371/journal.pone.0357049 (PMC13521393; doi:10.1371/journal.pone.0357049)
Supplement: Supplementary file 3 [file pone.0357049.s003.docx]

# **S3 File. Data Extraction Instrument.**

This tool will be refined and revised as needed during the data extraction process.

| **Field/Description** | **Extracted Data** |
| --- | --- |
| Title: |  |
| Author(s): List all authors of the study |  |
| Year of Publication: Year the study was published |  |
| Purpose of the study: Clear description of the study's objective(s) |  |
| Study design: Type of research design used (e.g., qualitative, quantitative, mixed-methods, observational, etc.) |  |
| Characteristics of the Target Population/Sample Size: Characteristics of the participants (e.g., age, gender, condition) and the sample size |  |
| Study Setting/Location: Where the study took place (e.g., country, hospital, community, rural, urban) |  |
| Study details: Summary of the study methodology, such as methods of data collection or intervention used |  |
| Key Findings: Include subcategories like theories/models, influencing factors, barriers and facilitators. |  |
| Study Recommendations: Recommendations provided by the authors (e.g., further research) |  |
| Contact with Authors: Whether authors were contacted for missing data and the outcome of that contact |  |
| Disagreements: Notes on any disagreements between reviewers and adjudication process (if applicable) |  |
| Additional comments |  |
