# Supplementary material for: Health-seeking behaviors of pregnant women with hypertensive disorders of pregnancy in low- and middle-income countries: A scoping review protocol
Source: PLoS One. 2026 Aug 27;21(8):e0357049. doi: 10.1371/journal.pone.0357049 (PMC13521393; doi:10.1371/journal.pone.0357049)
Supplement: Supplementary file 5 [file pone.0357049.s006.docx]

Dear Editor,

Thank you for the careful review of our protocol and for the constructive comments provided. We have addressed all points raised and revised the protocol where necessary. Below is a detailed, point-by-point response.

**Protocol Title:** Health-seeking behaviors of pregnant women with hypertensive disorders of pregnancy in low- and middle-income countries: A scoping review protocol.

| **Comments** | **Response** |
| --- | --- |
| **Reviewer 1** | |
| This protocol is submitted after the scoping review (identification and screening? is conducted). | Thank you for the question. The protocol was developed and submitted for publication prior to the initiation of the identification and screening phases. However, due to administrative processes related to manuscript submission there was a delay. Subsequently, the protocol was updated at the time of final submission to reflect that the true stage of the scoping review. The scoping review is currently ongoing, with the analysis, synthesis of findings, and discussion of results and limitations in progress. |
| Was there any date range for the publication identified and reviewed? | Thank you for this important question. We decided not to add date restrictions to the search strategy because our aim was to capture the full scope of available literature on the topic, regardless of the publication year. However, the dates of publication for all included studies will be reported to provide context regarding the evolution of the evidence base overtime in the scoping review. This information has been added on page 10 of manuscript. |
| Was the scoping review part of a bigger project? | The purpose of the scoping review provides a foundation to guide and inform my PhD research. This information has been added on page 7 of manuscript. |
| I think it would need an elaboration on the analysis and presentation planning. | Thank you for this suggestion. We have further elaborated on the data analysis and presentation plan to enhance clarity on page 11 and 12 of the manuscript. |
| Discussion on the protocol and how is the protocol compete with the current literature/how is its contribution to the literature is missing? | Thank you for this important question. We agree that further clarification regarding the contribution of this protocol to the existing literature is needed. This information has been added in the manuscript on page 7. |
| **Reviewer 2** | |
| Timelines; the search was conducted shortly after trial registration; but I am concerned that this protocol has been submitted at the time that the paper, apparently is due to be submitted; this suggests the protocol is being modified as the study is being completed which defeats the objective of a pre-specified protocol. | Thank you for raising this concern. We would like to clarify that the protocol was developed and submitted priori to guide the conduct of the scoping review. However, due to procedures associated with manuscript submission and academic timelines, the protocol was submitted for publication while the review was initiated. |
| The protocol states "two reviewers will review only relevant papers in full text, and one will extract data from all included papers." given the subject matter a second reviewer should independently extract data from a random 20% of included studies to ensure consistency, with any discrepancies resolved through discussion. | Thank you for this important recommendation. In our approach, a second reviewer was involved at multiple stages. The second reviewer independently reviewed the initial two papers (15%) during the data extraction phase to ensure alignment in the application of the extraction framework. Additionally, following completion of the full data extraction process, team members conducted a review of all extracted data to verify consistency and accuracy. Any discrepancies identified were resolved through discussion. This process has been included on page 12 of the manuscript. |
| The authors state "this search will concentrate exclusively on articles published in English, as the authors are fluent only in English." The authors correctly identify that Africa, Southeast Asia, and the Middle East have high prevalence rates but are precisely the regions where non-English publications are most likely to exist - this approach seems at odds with the focus of this review and is highly western-centric. | Thank you for this insightful comment. We acknowledge the concern regarding the limitation to English-language publications given that the regions may have relevant studies published in other languages. However, due to language constraints within the review team, the search was limited to English-language publications to ensure the accuracy in data extraction and interpretation. We recognize that this introduces a degree of selection bias and potentially limit the comprehensiveness of the review. Hence, we have acknowledged this as a limitation on page 9 of the manuscript. |
| The analysis plan requires much more clarity, for example; How will you distinguish barriers vs. facilitators from each other and from neutral factors? | Thank you for this valuable comment. We have included information to add more clarity regarding the analytical plan. This has been included on page 13-14 of the manuscript. |
| How will you categorize barriers (individual, health system, socioeconomic, cultural)? | Thank you for this important comment. This has been addressed on page 13 of the manuscript. |
| How will you handle contradictory findings across settings? | Thank you for this important comment. We recognize that contradictory findings may arise across different settings due to contextual variability. This information has been included on page 14 of the manuscript. |
| Will geographic/economic subgroup analysis be conducted? | Thank you for this important question. Yes, contextual information such as study setting, will be extracted and presented descriptively. Variations across geographic contexts will be explored narratively to highlight patterns, similarities, and differences in the reported. However, as a scoping review, a geographic and economic subgroup analyses will not be conducted in the form of statistical comparisons. This information has been added on page 13 of the manuscript. |
| The title and research question focus on pregnant women "with hypertensive disorders of pregnancy." This suggests women who already have an HDP diagnosis. However, early care-seeking (before diagnosis) is critical. Will the review include: Studies of general antenatal care-seeking (where HDP might be detected)? Studies of women at risk for HDP but not yet diagnosed? Only studies of women with confirmed HDP diagnosis? | Thank you for this important point of clarification. While the title and research question emphasize pregnant women with hypertensive disorders of pregnancy (HDP), the scope of this review is intentionally broader to capture the continuum of health-seeking behaviors relevant to HDP. Kindly, see page 8 of the manuscript. |
| **Reviewer 3** | |
| The authors should clarify the section regarding the definition of “health-seeking behavior.” While a general definition is provided, the manuscript would benefit from incorporating a clear conceptual framework to anchor both the definition and the analysis of this construct. | Thank you for this valuable suggestion. We agree that further conceptual clarity would strengthen the manuscript. This has been included on page 5 of the manuscript. |
| Additionally, it is unclear whether the authors are referring to appropriate (positive), inappropriate (negative) or both health-seeking behaviors, which could be further specified. | Thank you for this important point. This has been clarified on page 5 of the manuscript. |
| To have only one reviewer conducting data extraction may introduce risk of bias and reduce reliability, this issue could be address it in the limitations of the study. | Thank you for this important observation. We acknowledge that having a single reviewer conduct data extraction may introduce a risk of bias and could affect the reliability of the extracted data. To mitigate this, a second reviewer was involved at key stages of the process to ensure consistency, including review of initial extractions and verification of the final dataset. Kindly see page 12 of the manuscript. |
| Even though the restriction of the language of the studies to English is due to the fluency of the participants, this could introduce important risk of excluding substantial amount of evidence from regions where HDP burden is high, particularly given the focus on LMICs and inclusion of grey literature. The authors should discuss the potential impact on findings and consider strategies such as selective translation. | Thank you for this important comment. We fully acknowledge that restricting the review to English-language publications may result in the exclusion of relevant evidence, particularly from LMIC regions. This limitation may affect the comprehensiveness of the findings and introduce a degree of language bias. This has been acknowledged on page 9 of the manuscript. |
