# Supplementary material for: Health-seeking behaviors of pregnant women with hypertensive disorders of pregnancy in low- and middle-income countries: A scoping review protocol
Source: PLoS One. 2026 Aug 27;21(8):e0357049. doi: 10.1371/journal.pone.0357049 (PMC13521393; doi:10.1371/journal.pone.0357049)
Supplement: Peer review history

# Peer review: Decision Letter 0

**DOI:** 10.1371/journal.pone.0357049.r001

**Authors:** Khin Thet Wai

6 May 2026

Dear Dr. Adam Al-Hassan,

plosone@plos.org. When you're ready to submit your revision, log on to https://www.editorialmanager.com/pone/ and select the 'Submissions Needing Revision' folder to locate your manuscript file.

- A letter that responds to each point raised by the academic editor and reviewer(s). You should upload this letter as a separate file labeled 'Response to Reviewers'.
- A marked-up copy of your manuscript that highlights changes made to the original version. You should upload this as a separate file labeled 'Revised Manuscript with Track Changes'.
- An unmarked version of your revised paper without tracked changes. You should upload this as a separate file labeled 'Manuscript'.

If you would like to make changes to your financial disclosure, please include your updated statement in your cover letter. Guidelines for resubmitting your figure files are available below the reviewer comments at the end of this letter.

If applicable, we recommend that you deposit your laboratory protocols in protocols.io to enhance the reproducibility of your results. Protocols.io assigns your protocol its own identifier (DOI) so that it can be cited independently in the future. For instructions see: https://journals.plos.org/plosone/s/submission-guidelines#loc-laboratory-protocols. Additionally, PLOS ONE offers an option for publishing peer-reviewed Lab Protocol articles, which describe protocols hosted on protocols.io. Read more information on sharing protocols at https://plos.org/protocols?utm_medium=editorial-email&utm_source=authorletters&utm_campaign=protocols.

As the corresponding author, your ORCID iD is verified in the submission system and will appear in the published article. PLOS supports the use of ORCID, and we encourage all coauthors to register for an ORCID iD and use it as well. Please encourage your coauthors to verify their ORCID iD within the submission system before final acceptance, as unverified ORCID iDs will not appear in the published article. *Only*  the individual author can complete the verification step; PLOS staff *cannot*  verify ORCID iDs on behalf of authors.

We look forward to receiving your revised manuscript.

Kind regards,

Khin Thet Wai, MBBS, MPH, MA

Academic Editor

PLOS One

Journal Requirements:

When submitting your revision, we need you to address these additional requirements.

1.Please ensure that your manuscript meets PLOS ONE's style requirements, including those for file naming. The PLOS ONE style templates can be found at

https://journals.plos.org/plosone/s/file?id=wjVg/PLOSOne_formatting_sample_main_body.pdf and

https://journals.plos.org/plosone/s/file?id=ba62/PLOSOne_formatting_sample_title_authors_affiliations.pdf

2. If the reviewer comments include a recommendation to cite specific previously published works, please review and evaluate these publications to determine whether they are relevant and should be cited. There is no requirement to cite these works unless the editor has indicated otherwise.

3. Please review your reference list to ensure that it is complete and correct. If you have cited papers that have been retracted, please include the rationale for doing so in the manuscript text, or remove these references and replace them with relevant current references. Any changes to the reference list should be mentioned in the rebuttal letter that accompanies your revised manuscript. If you need to cite a retracted article, indicate the article’s retracted status in the References list and also include a citation and full reference for the retraction notice.

Additional Editor Comments:

Please do minor revisions as required.

[Note: HTML markup is below. Please do not edit.]

Reviewers' comments:

Reviewer's Responses to Questions

**Comments to the Author**

1. Does the manuscript provide a valid rationale for the proposed study, with clearly identified and justified research questions?

Reviewer #1: Yes

Reviewer #2: Yes

Reviewer #3: Yes

**********

2. Is the protocol technically sound and planned in a manner that will lead to a meaningful outcome and allow testing the stated hypotheses?

Reviewer #1: Partly

Reviewer #2: No

Reviewer #3: Yes

**********

3. Is the methodology feasible and described in sufficient detail to allow the work to be replicable?

Reviewer #1: Yes

Reviewer #2: No

Reviewer #3: Yes

**********

4. Have the authors described where all data underlying the findings will be made available when the study is complete?

The PLOS Data policy

Reviewer #1: Yes

Reviewer #2: Yes

Reviewer #3: Yes

**********

5. Is the manuscript presented in an intelligible fashion and written in standard English?

Reviewer #1: Yes

Reviewer #2: Yes

Reviewer #3: Yes

**********

Please use the space provided to explain your answers to the questions above and, if applicable, provide comments about issues authors must address before this protocol can be accepted for publication. You may also include additional comments for the author, including concerns about research or publication ethics.

You may also provide optional suggestions and comments to authors that they might find helpful in planning their study.

Reviewer #1: Dear Editor,

Thank you very much for the opportunity to review this protocol, this protocol is submitted after the scoping review (identification and screening? is conducted)

Couple of minor questions:

- Was there any date range for the publication identified and reviewed?

- Was the scoping review part of a bigger project?

- I think it would need an elaboration on the analysis and presentation planning

- Discussion on the protocol and how is the protocol compete with the current literature/how is its contribution to the literature is missing?

Reviewer #2: The authors propose a scoping review examining health-seeking behaviors of pregnant women with hypertensive disorders of pregnancy (HDP) in low- and middle-income countries. This is an important and timely research question that addresses a significant gap in the literature. As a clinician working in this field, I recognize the value of understanding barriers and facilitators to care-seeking in resource-limited settings, where the burden of HDP-related morbidity and mortality remains disproportionately high.

However, I have several concerns, primarily:

- Timelines; the search was conducted shortly after trial registration; but I am concerned that this protocol has been submitted at the time that the paper, apparently is due to be submitted; this suggests the protocol is being modified as the study is being completed which defeats the objective of a pre-specified protocol.

- The protocol states "two reviewers will review only relevant papers in full text, and one will extract data from all included papers." given the subject matter a second reviewer should independently extract data from a random 20% of included studies to ensure consistency, with any discrepancies resolved through discussion.

- The authors state "this search will concentrate exclusively on articles published in English, as the authors are fluent only in English." The authors correctly identify that Africa, Southeast Asia, and the Middle East have high prevalence rates but are precisely the regions where non-English publications are most likely to exist - this approach seems at odds with the focus of this review and is highly western-centric.

- The analysis plan requires much more clarity, for example;

How will you distinguish barriers vs. facilitators from each other and from neutral factors?

How will you categorize barriers (individual, health system, socioeconomic, cultural)?

How will you handle contradictory findings across settings?

Will geographic/economic subgroup analysis be conducted?

- The title and research question focus on pregnant women "with hypertensive disorders of pregnancy." This suggests women who already have an HDP diagnosis. However, early care-seeking (before diagnosis) is critical. Will the review include:

Studies of general antenatal care-seeking (where HDP might be detected)?

Studies of women at risk for HDP but not yet diagnosed?

Only studies of women with confirmed HDP diagnosis?

Reviewer #3: This protocol addresses an extremely important and unexplored topic within maternal health: the health-seeking behaviors of pregnant women with hypertensive disorders of pregnancy (HDP) in low- and middle-income countries (LMICs). This topic is clinically significant and aligns with global health priorities, particularly related with inequities in maternal health.

The justification for conducting a scoping review is well-articulated. The inclusion of multiple study designs is appropriate given the behavioral and sociocultural nature of the research question. Furthermore, the inclusion of grey literature is a methodological strength, particularly in LMIC contexts where non-indexed evidence may be substantial. The registration into OSF, the use of Joanna Briggs Institute (JBI) methodology and PRISMA-ScR reporting standards is methodologically appropriate for a scoping review.

The authors should clarify the section regarding the definition of “health-seeking behavior.” While a general definition is provided, the manuscript would benefit from incorporating a clear conceptual framework to anchor both the definition and the analysis of this construct. Additionally, it is unclear whether the authors are referring to appropriate (positive), inappropriate (negative) or both health-seeking behaviors, which could be further specified.

To have only one reviewer conducting data extraction may introduce risk of bias and reduce reliability, this issue could be address it in the limitations of the study.

Even though the restriction of the language of the studies to English is due to the fluency of the participants, this could introduce important risk of excluding substantial amount of evidence from regions where HDP burden is high, particularly given the focus on LMICs and inclusion of grey literature. The authors should discuss the potential impact on findings and consider strategies such as selective translation.

**********

what does this mean?). If published, this will include your full peer review and any attached files.

If you choose “no”, your identity will remain anonymous but your review may still be made public.

**Do you want your identity to be public for this peer review?**  For information about this choice, including consent withdrawal, please see our Privacy Policy

Reviewer #1: No

Reviewer #2: No

Reviewer #3: No

**********

[NOTE: If reviewer comments were submitted as an attachment file, they will be attached to this email and accessible via the submission site. Please log into your account, locate the manuscript record, and check for the action link "View Attachments". If this link does not appear, there are no attachment files.]

To ensure your figures meet our technical requirements, please review our figure guidelines: https://journals.plos.org/plosone/s/figures

You may also use PLOS’s free figure tool, NAAS, to help you prepare publication quality figures: https://journals.plos.org/plosone/s/figures#loc-tools-for-figure-preparation.

NAAS will assess whether your figures meet our technical requirements by comparing each figure against our figure specifications.

# Author comment: Author response to Decision Letter 1

**DOI:** 10.1371/journal.pone.0357049.r002

14 Jun 2026

Dear Editor,

Thank you for the careful review of our protocol and for the constructive comments provided. We have addressed all points raised and revised the protocol where necessary. Below is a detailed, point-by-point response.

Protocol Title: Health-seeking behaviors of pregnant women with hypertensive disorders of pregnancy in low- and middle-income countries: A scoping review protocol.

Comments Response

Reviewer 1: This protocol is submitted after the scoping review (identification and screening? is conducted).

Authors' response: Thank you for the question. The protocol was developed and submitted for publication prior to the initiation of the identification and screening phases. However, due to administrative processes related to manuscript submission there was a delay. Subsequently, the protocol was updated at the time of final submission to reflect that the true stage of the scoping review. The scoping review is currently ongoing, with the analysis, synthesis of findings, and discussion of results and limitations in progress.

Was there any date range for the publication identified and reviewed?

Authors' response: Thank you for this important question. We decided not to add date restrictions to the search strategy because our aim was to capture the full scope of available literature on the topic, regardless of the publication year. However, the dates of publication for all included studies will be reported to provide context regarding the evolution of the evidence base overtime in the scoping review. This information has been added on page 10 of manuscript.

Was the scoping review part of a bigger project?

Authors' response: The purpose of the scoping review provides a foundation to guide and inform my PhD research. This information has been added on page 7 of manuscript.

I think it would need an elaboration on the analysis and presentation planning.

Authors' response: Thank you for this suggestion. We have further elaborated on the data analysis and presentation plan to enhance clarity on page 11 and 12 of the manuscript.

Discussion on the protocol and how is the protocol compete with the current literature/how is its contribution to the literature is missing?

Authors' response: Thank you for this important question. We agree that further clarification regarding the contribution of this protocol to the existing literature is needed. This information has been added in the manuscript on page 7.

Reviewer 2

Timelines; the search was conducted shortly after trial registration; but I am concerned that this protocol has been submitted at the time that the paper, apparently is due to be submitted; this suggests the protocol is being modified as the study is being completed which defeats the objective of a pre-specified protocol.

Authors' response: Thank you for raising this concern. We would like to clarify that the protocol was developed and submitted priori to guide the conduct of the scoping review. However, due to procedures associated with manuscript submission and academic timelines, the protocol was submitted for publication while the review was initiated.

The protocol states "two reviewers will review only relevant papers in full text, and one will extract data from all included papers." given the subject matter a second reviewer should independently extract data from a random 20% of included studies to ensure consistency, with any discrepancies resolved through discussion.

Authors' response: Thank you for this important recommendation. In our approach, a second reviewer was involved at multiple stages. The second reviewer independently reviewed the initial two papers (15%) during the data extraction phase to ensure alignment in the application of the extraction framework. Additionally, following completion of the full data extraction process, team members conducted a review of all extracted data to verify consistency and accuracy. Any discrepancies identified were resolved through discussion. This process has been included on page 12 of the manuscript.

The authors state "this search will concentrate exclusively on articles published in English, as the authors are fluent only in English." The authors correctly identify that Africa, Southeast Asia, and the Middle East have high prevalence rates but are precisely the regions where non-English publications are most likely to exist - this approach seems at odds with the focus of this review and is highly western-centric.

Authors' response: Thank you for this insightful comment. We acknowledge the concern regarding the limitation to English-language publications given that the regions may have relevant studies published in other languages. However, due to language constraints within the review team, the search was limited to English-language publications to ensure the accuracy in data extraction and interpretation. We recognize that this introduces a degree of selection bias and potentially limit the comprehensiveness of the review. Hence, we have acknowledged this as a limitation on page 9 of the manuscript.

The analysis plan requires much more clarity, for example; How will you distinguish barriers vs. facilitators from each other and from neutral factors?

Authors' response: Thank you for this valuable comment. We have included information to add more clarity regarding the analytical plan. This has been included on page 13-14 of the manuscript.

How will you categorize barriers (individual, health system, socioeconomic, cultural)?

Authors' response: Thank you for this important comment. This has been addressed on page 13 of the manuscript.

How will you handle contradictory findings across settings?

Authors' response: Thank you for this important comment. We recognize that contradictory findings may arise across different settings due to contextual variability. This information has been included on page 14 of the manuscript.

Will geographic/economic subgroup analysis be conducted?

Authors' response: Thank you for this important question. Yes, contextual information such as study setting, will be extracted and presented descriptively. Variations across geographic contexts will be explored narratively to highlight patterns, similarities, and differences in the reported. However, as a scoping review, a geographic and economic subgroup analyses will not be conducted in the form of statistical comparisons. This information has been added on page 13 of the manuscript.

The title and research question focus on pregnant women "with hypertensive disorders of pregnancy." This suggests women who already have an HDP diagnosis. However, early care-seeking (before diagnosis) is critical. Will the review include:

Studies of general antenatal care-seeking (where HDP might be detected)?

Studies of women at risk for HDP but not yet diagnosed?

Only studies of women with confirmed HDP diagnosis?

Authors' response: Thank you for this important point of clarification. While the title and research question emphasize pregnant women with hypertensive disorders of pregnancy (HDP), the scope of this review is intentionally broader to capture the continuum of health-seeking behaviors relevant to HDP. Kindly, see page 8 of the manuscript.

Reviewer 3

The authors should clarify the section regarding the definition of “health-seeking behavior.” While a general definition is provided, the manuscript would benefit from incorporating a clear conceptual framework to anchor both the definition and the analysis of this construct.

Authors' response: Thank you for this valuable suggestion. We agree that further conceptual clarity would strengthen the manuscript. This has been included on page 5 of the manuscript.

Additionally, it is unclear whether the authors are referring to appropriate (positive), inappropriate (negative) or both health-seeking behaviors, which could be further specified.

Authors' response: Thank you for this important point. This has been clarified on page 5 of the manuscript.

To have only one reviewer conducting data extraction may introduce risk of bias and reduce reliability, this issue could be address it in the limitations of the study.

Authors' response: Thank you for this important observation. We acknowledge that having a single reviewer conduct data extraction may introduce a risk of bias and could affect the reliability of the extracted data. To mitigate this, a second reviewer was involved at key stages of the process to ensure consistency, including review of initial extractions and verification of the final dataset. Kindly see page 12 of the manuscript.

Even though the restriction of the language of the studies to English is due to the fluency of the participants, this could introduce important risk of excluding substantial amount of evidence from regions where HDP burden is high, particularly given the focus on LMICs and inclusion of grey literature. The authors should discuss the potential impact on findings and consider strategies such as selective translation.

Authors' response: Thank you for this important comment. We fully acknowledge that restricting the review to English-language publications may result in the exclusion of relevant evidence, particularly from LMIC regions. This limitation may affect the comprehensiveness of the findings and introduce a degree of language bias. This has been acknowledged on page 9 of the manuscript.

Attachment Submitted filename: Response to reviewers.docx

# Peer review: Decision Letter 1

**DOI:** 10.1371/journal.pone.0357049.r003

**Authors:** Khin Thet Wai

11 Aug 2026

Health-seeking behaviors of pregnant women with hypertensive disorders of pregnancy in low- and middle-income countries: A scoping review protocol

PONE-D-25-37450R1

Dear Dr. Adam Al-Hassan,

We’re pleased to inform you that your manuscript has been judged scientifically suitable for publication and will be formally accepted for publication once it meets all outstanding technical requirements.

Within one week, you’ll receive an e-mail detailing the required amendments. When these have been addressed, you’ll receive a formal acceptance letter and your manuscript will be scheduled for publication.

An invoice will be generated when your article is formally accepted. Please note, if your institution has a publishing partnership with PLOS and your article meets the relevant criteria, all or part of your publication costs will be covered. Please make sure your user information is up-to-date by logging into Editorial Manager at Editorial Manager® and clicking the ‘Update My Information' link at the top of the page. For questions related to billing, please contact billing support.

If your institution or institutions have a press office, please notify them about your upcoming paper to help maximize its impact. If they’ll be preparing press materials, please inform our press team as soon as possible -- no later than 48 hours after receiving the formal acceptance. Your manuscript will remain under strict press embargo until 2 pm Eastern Time on the date of publication. For more information, please contact onepress@plos.org.

Kind regards,

Khin Thet Wai, MBBS, MPH, MA

Academic Editor

PLOS One

Additional Editor Comments (optional):

All comments are adequately addressed.

Reviewers' comments:

Reviewer's Responses to Questions

**Comments to the Author**

1. Does the manuscript provide a valid rationale for the proposed study, with clearly identified and justified research questions?

Reviewer #3: Yes

**********

2. Is the protocol technically sound and planned in a manner that will lead to a meaningful outcome and allow testing the stated hypotheses?

Reviewer #3: Partly

**********

3. Is the methodology feasible and described in sufficient detail to allow the work to be replicable?

Reviewer #3: Yes

**********

4. Have the authors described where all data underlying the findings will be made available when the study is complete?

The PLOS Data policy

Reviewer #3: Yes

**********

5. Is the manuscript presented in an intelligible fashion and written in standard English?

Reviewer #3: Yes

**********

Please use the space provided to explain your answers to the questions above and, if applicable, provide comments about issues authors must address before this protocol can be accepted for publication. You may also include additional comments for the author, including concerns about research or publication ethics.

You may also provide optional suggestions and comments to authors that they might find helpful in planning their study.

Reviewer #3: I thank the authors for their responsive revision. My three comments have been adequately addressed.

1. Conceptual framework. The construct is now based in the socio-ecological model (SEM), which now resolves my concern. As an optional refinement, the authors could make the correspondence between SEM levels and the three analytic domains explicit in the Data analysis and presentation section, so the framework is fully operationalized.

2. Appropriate vs. inappropriate health-seeking behavior. The revised text now explicitly differentiates positive behaviors (timely antenatal attendance, prompt care-seeking) from negative ones (delayed presentation, reliance on non-formal care). This resolves the ambiguity I had noted.

3. Single-reviewer data extraction. The authors clarified that a second reviewer verified the initial extractions and reviewed all extracted data upon completion, with discrepancies resolved through discussion. This is a reasonable mitigation, though it falls short of the random 20% re-extraction requested by Reviewer 2: verification covered 15% of studies (the first two extracted, not a random sample). For a scoping review this is acceptable, but I would still encourage the authors to state the single-extractor design as a limitation, as originally suggested. If the editor prefers a stricter standard, that would be aligned with Reviewer 2's original request for a random 20% re-extraction.

Overall, this is a methodologically sound, clearly written protocol addressing a relevant evidence gap in maternal health in LMICs. The remaining points are minor and editorial and do not, in my view, require a further round of review. I recommend acceptance.

I note that other reviewers raised concerns about protocol timing relative to the conduct of the review. I regard this as a matter for editorial judgment, not affecting my assessment of methodological soundness.

**********

what does this mean?). If published, this will include your full peer review and any attached files.

If you choose “no”, your identity will remain anonymous but your review may still be made public.

**Do you want your identity to be public for this peer review?**  For information about this choice, including consent withdrawal, please see our Privacy Policy

Reviewer #3: No

**********

# Editor report: Acceptance letter

**DOI:** 10.1371/journal.pone.0357049.r004

**Authors:** Khin Thet Wai

PONE-D-25-37450R1

PLOS One

Dear Dr. Adam Al-Hassan,

I'm pleased to inform you that your manuscript has been deemed suitable for publication in PLOS One. Congratulations! Your manuscript is now being handed over to our production team.

At this stage, our production department will prepare your paper for publication. This includes ensuring the following:

* All references, tables, and figures are properly cited

* All relevant supporting information is included in the manuscript submission,

* There are no issues that prevent the paper from being properly typeset

You will receive further instructions from the production team, including instructions on how to review your proof when it is ready. Please keep in mind that we are working through a large volume of accepted articles, so please give us a few days to review your paper and let you know the next and final steps.

Lastly, if your institution or institutions have a press office, please let them know about your upcoming paper now to help maximize its impact. If they'll be preparing press materials, please inform our press team within the next 48 hours. Your manuscript will remain under strict press embargo until 2 pm Eastern Time on the date of publication. For more information, please contact onepress@plos.org.

You will receive an invoice from PLOS for your publication fee after your manuscript has reached the completed accept phase. If you receive an email requesting payment before acceptance or for any other service, this may be a phishing scheme. Learn how to identify phishing emails and protect your accounts at https://explore.plos.org/phishing.

If we can help with anything else, please email us at customercare@plos.org.

Thank you for submitting your work to PLOS One and supporting open access.

Kind regards,

PLOS ONE Editorial Office Staff

on behalf of

Dr. Khin Thet Wai

Academic Editor

PLOS One
